# Adsorption- and Displacement-Based Approaches for the Removal of Protein-Bound Uremic Toxins

**DOI:** 10.3390/toxins15020110

**Published:** 2023-01-28

**Authors:** Flávia S. C. Rodrigues, Mónica Faria

**Affiliations:** 1 Laboratory of Physics of Materials and Emerging Technologies (LaPMET), Center of Physics and Engineering of Advanced Materials (CeFEMA), Instituto Superior Técnico, Universidade de Lisboa, Av. Rovisco Pais, 1049-001 Lisbon, Portugal; 2 Chemical Engineering Department, Instituto Superior Técnico, Universidade de Lisboa, Av. Rovisco Pais, 1049-001 Lisbon, Portugal

**Keywords:** end-stage renal disease, hemodialysis, protein-bound uremic toxins, uremic toxins, indoxyl sulfate, p-cresyl sulfate, mixed matrix membranes, adsorption, competitive binding, displacement technology

## Abstract

End-stage renal disease (ESRD) patients rely on renal replacement therapies to survive. Hemodialysis (HD), the most widely applied treatment, is responsible for the removal of excess fluid and uremic toxins (UTs) from blood, particularly those with low molecular weight (MW < 500 Da). The development of high-flux membranes and more efficient treatment modes, such as hemodiafiltration, have resulted in improved removal rates of UTs in the middle molecular weight range. However, the concentrations of protein-bound uremic toxins (PBUTs) remain essentially untouched. Due to the high binding affinity to large proteins, such as albumin, PBUTs form large complexes (MW > 66 kDa) which are not removed during HD and their accumulation has been strongly associated with the increased morbidity and mortality of patients with ESRD. In this review, we describe adsorption- and displacement-based approaches currently being studied to enhance the removal of PBUTs. The development of mixed matrix membranes (MMMs) with selective adsorption properties, infusion of compounds capable of displacing UTs from their binding site on albumin, and competitive binding membranes show promising results, but the road to clinical application is still long, and further investigation is required.

## 1. Introduction

Recent studies suggest that 9.1% to 13.4% of the worldwide population has chronic kidney disease (CKD) [1,2]. More than 100 different uremic retention solutes, also referred to as uremic toxins (UTs), are known to accumulate in CKD patients due to impaired renal clearance. The accumulation of UTs in the blood further deteriorates renal function which can lead to irreversible kidney failure, a condition known as an end-stage renal disease (ESRD). To survive, there are only two options for patients diagnosed with ESRD: renal replacement therapy (RRT) and transplantation. Even though transplantation is the most effective, it is limited by the scarcity of organ donors and therefore most patients rely on RRTs such as hemodialysis (HD). HD is an extracorporeal circulation practice for blood purification and is typically performed in four-hour sessions thrice weekly—a procedure which must continue until either transplantation or death. The main component of the HD circuit is the hemodialyzer, also known as the artificial kidney, which is composed of semipermeable polymer hollow fiber membranes responsible for the removal of excess fluid and UTs from the blood of ESRD patients [3].

UTs are metabolites that are efficiently removed or metabolized by the healthy kidney, but in patients with ESRD, accumulate in the blood reaching concentrations which ultimately affect the normal function of not only cells and tissues but almost every organ system—a phenomenon commonly referred to as the uremic syndrome [4,5]. The European Uremic Toxin work group (EUTox) has identified more than 140 different UTs and has classified them into three different categories: (i) free water-soluble low molecular weight molecules (LMWMs), (ii) middle molecules, and (iii) protein-bound uremic toxins (PBUTs) [3,6]. LMWMs are defined as small solutes with molecular weights (MWs) below 500 Da. Urea, creatinine, and uric acid are three well-known examples of LMWMs. Middle molecules have an MW between 500 and 60,000 Da. β_2_-microglobulin (β_2_m) is the surrogate marker of middle molecules and the most well studied. PBUTs have a very high affinity for plasma proteins, especially to human serum albumin (HSA), and circulate in the blood in two different modes: bound or free (unbound). In their free form, most PBUTs have MWs smaller or equal to 500 Da, but when bound to HSA, form albumin-toxin complexes with very large MWs (>66.5 kDa) [7]. Indoxyl sulfate (IS) and p-cresyl sulfate (pCS) are PBUTs which have been thoroughly investigated in the last decade [6]. It is known that PBUTs accumulate both in the blood and tissues of patients [8] and that this accumulation contributes greatly to the progression of CKD [9]. In general, PBUTs are referred to as the most “problematic” group of UTs due to the challenges faced in their removal as well as the deleterious effects they have on ESRD patients.

During a typical HD session, the UT-concentrated blood is pumped through a hemodialyzer where excess water and UTs capable of crossing the semipermeable membranes, move from the blood side into the dialysate stream. The purified blood is returned to the patient while the spent dialysate is recycled in a nearby reverse osmosis system. Current hemodialyzers are composed of polymeric hollow fiber membranes and operate under the inside-out filtration (IOF) mode where blood flows in the lumen and dialysate flows on the outer side of the hollow fibers. The blood flow rate varies between 300 and 550 mL/min, and the transmembrane pressure (TMP) is regulated between 0 (no forced convection) and 300 mmHg (forced convection) by pressure valves present in the system. The dialysate flows counter-current to blood, at a flow rate of approximately 500 mL/min, and the pressure in the dialysate compartment is normally regulated so that it is lower than the pressure in the blood compartment to avoid back filtration [10].

The two main mass transport mechanisms involved in the removal of UTs and excess water from the blood of ESRD patients to the dialysate during HD are diffusion and convection [11]. Diffusion of solutes through a semipermeable membrane is characterized by the movement of solutes from a location of high concentration (usually the blood compartment) to a location of low concentration (usually the dialysate compartment), where the driving force is the concentration gradient across the membrane. Transport of LMWMs from the blood to the dialysate is commonly performed by diffusion where the TMP between the blood and dialysate compartments is maintained at 0 mmHg [12,13]. Convection is the forced movement of fluid and solutes across the HD membrane by a pressure gradient (TMP) and it is primarily responsible for removing excess fluid and solutes with higher MWs from the blood of ESRD patients [12].

In the past, HD membranes were generally classified according to their composition (cellulosic or synthetic) and water permeability [11]. Presently, membranes are classified as suitable for low-flux hemodialysis (LFHD) or high-flux hemodialysis (HFHD) according to the ultrafiltration coefficient (K_UF_), which is defined as the amount of water that crosses the membrane per unit of time and per mmHg of TMP in an in vitro setting using animal blood (frequently bovine blood) [14]. Values being lower or higher than 12 mL/h/mmHg distinguish LFHD from HFHD, respectively [11]. Other parameters used in the classification of more modern hemodialyzers are: (i) the capability of removing middle MW molecules, such as β_2_m, (ii) permeability to HSA, also referred to as protein or albumin leakage, and (iii) molecular weight cut-off (MWCO), which is defined as the MW of a specific solute which is at least 90% retained by the membrane [15].

The average MWCO of commercial HD membranes is approximately 15 kDa but recently, two new classes of membranes have been proposed: membranes for medium cut-off hemodialysis (MCO-HD) which exhibit MWCO values slightly lower than the MW of HSA (66.5 kDa) and high cut-off hemodialysis (HCO-HD) membranes which have an MWCO of approximately 200 kDa [11,16,17,18]. MCO-HD membranes offer an improved clearance of UTs with an MW range between 15 and 45 kDa and protein leakage is prevented due to the MWCO being below the MW of HSA [17]. HCO-HD membranes show improvement in the clearance of middle molecules with higher MWs as well as the clearance of PBUTs; however, they are associated with substantial albumin leakage varying being between 9 and 23 g per HD session. HCO-HD membranes are not suitable for continuous use but are recommended for acute applications [18].

Most of the HD membranes available today are made of synthetic polymers such as polysulfone (PS), polyvinylpyrrilidone (PVP), and polyamide [19]. The PS-based dialyzers commercialized by Fresenius Medical Care AG and Co. KGaA (Bad Homburg, Germany) are suitable for both LFHD and HFHD covering a wide range of K_UF_ values between 8 and 73 mL/h/mmHg and are known for their efficient removal of LMWMs as well as several middle molecules [20]. The PES/PVP-polyamide blend hemodialyzers, commercialized by Baxter International, Inc. (Deerfield, IL, USA), are intended for both LFHD and HFHD with K_UF_ values ranging between 10 and 85 mL/h/mmHg [21,22]. The Nipro Corporation (Osaka, Japan) is one of the rare companies which continues to invest in nonsynthetic hemodialyzers. The Sureflux™ and Salacea™ cellulose triacetate (CTA) hemodialyzers have K_UF_ values ranging from 3 to 87 mL/h/mmHg and from 61 to 87 mL/h/mmHg, respectively, making Salacea™ suitable for HFHD and Sureflux™ appropriate for both LFHD and HFHD [23,24].

When operated under forced convection, current hemodialyzers are efficient in the removal of excess fluid from ESRD patients while retaining blood cells and other vital blood components (proteins, platelets, etc.). In contrast, they are far from efficient in terms of efficiently detoxifying blood as they present very low clearances of middle molecules as well as the bound fraction of PBUTs [25,26], and in general, any UTs with MWs > 15 kDa [5,11]. IS and pCS, two well-known PBUTs, are known to circulate in the blood almost completely bound to HSA achieving binding percentages of ~98% and ~95%, respectively [25]. Studies have proved that the accumulation of high concentrations of IS and pCS in the blood of ESRD patients contributes to increased expression of inflammatory genes, vascular damage, and cardiovascular disease [27,28]. Other PBUTs such as hippuric acid (HA), indole-3-acetic acid (IAA), and 3-carboxyl4-methil-5-propyl-furanpropionic acid (CMPF), have binding percentages of 48%, 94%, and ~100%, respectively [25]. The primary binding sites for these PBUTs are Sudlow’s site II of the HSA molecule, with the exception of CMPF which binds to site I [3,29].

In the last decade, many strategies have been proposed to decrease the accumulation of PBUTs in the bloodstream of CKD and ESRD patients [3] including (i) maintenance of residual kidney function [30]; (ii) limitation of PBUTs generated in the colon [31]; (iii) oral administration of charcoal adsorbents such as AST-120 (Kremezin) [32]; (iv) development of more efficient HD membranes or membrane-based renal replacement therapies [33]; and (v) use of displacement technology coupled with current RRTs [34].

This review focuses on the last two: adsorption- and displacement-based approaches currently being investigated towards enhanced removal of PBUTs. The research focused on the development of adsorptive HD membranes, the infusion of protein-binding competitors into the bloodstream of patients undergoing HD, and the development of competitive binding membranes which are still in the early stages of development are discussed. The studies reported in this review are in no way definitive and will certainly be expanded as research progresses. They do, however, provide insight into the interdisciplinary research being conducted across clinical medicine, material scientists, and research engineers which is much needed to bring the often neglected problem of PBUTs into the limelight.

## 2. Adsorptive Membranes

**Figure 1:**
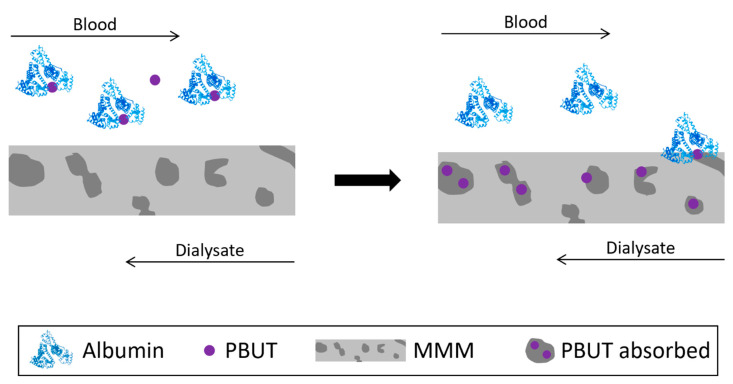
Schematical representation of the removal of PBUTs by adsorptive membranes.

Adsorptive membranes are generally prepared by mixing adsorptive filler particles with traditional polymer materials and exhibit the dual function of adsorption and membrane separation [35,36]. Figure 1 schematically shows the concept behind adsorptive membranes where nanoparticles of different nature (activated carbon, zeolites, metal-organic frameworks, etc.) are incorporated into the polymer matrix of the membrane to produce mixed matrix membranes (MMMs) capable of adsorbing PBUTs.

**Figure 2:**
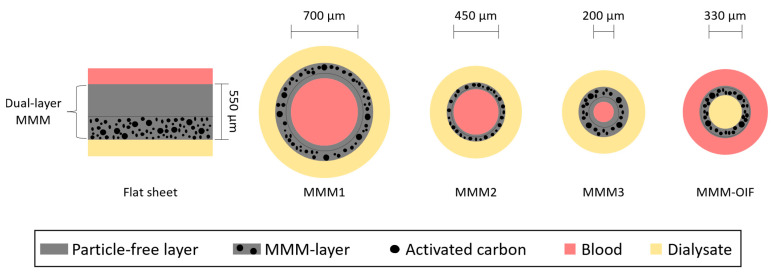
Flat sheet and hollow fiber dual-layer PES/PVP-AC mixed matrix membranes.

During the last decade, dual-layer MMMs, composed of a polymer polyethersulfone/polyvinylpyrrolidone (PES/PVP) blend with activated carbon (AC) particles, have been extensively researched. Development of the dual-layer MMMs began in 2012 by Tijink et al. [37] with the production of the membranes in the form of flat sheets followed shortly by three different types of hollow fibers where blood circulated on the inside of the fibers and dialysate on the outside [38,39,40]. More recently, Beek et al. [41], prepared dual-layer hollow fibers intended for outside-in filtration (OIF) or extraluminal flow therapy, where the blood flows on the outside of the hollow fibers while dialysate flows in the lumen. Figure 2 shows a scheme of the five different PES/PVP-AC MMMs developed over the last decade.

The first MMMs were produced in the form of flat sheets and were prepared by incorporating activated carbon (AC) into a mixture of polyethersulfone (PES) and polyvinylpyrrolidone (PVP) [37]. AC is considered one of the most versatile adsorbents because of its large surface area, microporous structure, high adsorption capacity, and variable surface chemical composition. It has been used extensively to purify liquids and gases in a variety of applications, including drinking water, food and beverage processing, odor removal, industrial pollution control, etc. However, there are risks associated with the use of AC for blood perfusion applications such as poor hemocompatibility, fine particle release into the bloodstream, and plasma protein adsorption [42]. Tijink et al. [37] prepared three flat sheet membranes: (i) a particle-free PES/PVP dense membrane (without AC); (ii) a single-layer MMMs, which contains AC particles dispersed throughout a porous PES/PVP matrix; and (iii) a dual-layer MMMs, which contains AC particles in the porous membrane matrix and no AC particle in the thin dense layer which contacts blood [37]. The three membranes together with a sample of pure AC were incubated separately (under static conditions), in volumes of plasma taken from ESRD patients for 4 h, to evaluate the adsorption of hippuric acid (HA) [37]. Results showed that in the pure AC sample, the single and dual-layer MMMs adsorbed over 80% of HA present in the plasma. In contrast, the particle-free membrane did not absorb any HA from the plasma [37]. These results indicated that the presence of AC on its own or incorporated into PES/PVP mixtures promoted the adsorption of HA.

Tijink et al. [38], developed the first generation of PES/PVP-AC-based hollow fiber membranes, named MMM1, intended for inside-out filtration (IOF), i.e., blood/plasma circulates in the lumen of the hollow fibers while dialysate flows on the outside of the fibers. The lumen of the hollow fibers (the surface that is in direct contact with blood) is composed of a thin dense layer composed solely of PES and PVP which does not contain any particles of AC—this prevents direct contact between blood and AC. The outer layer is much thicker and has a porous structure which is composed of PES, PVP, and dispersed AC particles. The MMM1 was tested in two different conditions of plasma and dialysate circulation: one where transport of solutes from the plasma to dialysate occurred only by diffusion and another where a TMP was applied so that transport occurred via forced convection. Separate volumes of healthy human plasma were spiked with pCS, IS and HA, and after incubation, the bound percentage of each toxin was 90%, 87%, and 38%, respectively. Results showed that after 6 h of plasma circulation through the MMM1 where only diffusion occurred, 2.3 mg of pCS, 3.6 mg of IS, and 14 mg of HA per gram of membrane were removed, and 100% of the HSA was retained. In the experiment where convection took place, results showed that after 4 h, 2.7 mg of pCS, 12.9 mg of IS, and 15 mg of HA per gram of membrane were removed; however, there was evidence of protein leaking as the membrane did not retain all the HSA [38]. The K_UF_ of the MMM1 was 78 mL/h/m^2^/mmHg making it suitable for HFHD, but the internal diameter was approximately 700 µm which is more than double the diameter of membranes currently used in clinical practices (~200 µm), making them unsuitable for clinical application. In order to overcome this limitation, Pavlenko et al. [39], developed the second generation of PES/PVP-AC-based hollow fibers (MMM2) with internal diameters of approximately 450 µm. The membranes exhibited a K_UF_ value of 3.37 mL/h/m^2^/mmHg which makes them suitable for LFHD and no HSA leakage was detected throughout the permeation experiments. To evaluate the removal of PBUTs by the MMM2, healthy human plasma was spiked with IS and pCS at a concentration of 40 mg/L and crossflow filtration experiments were carried out at a TMP of 0 mmHg (no forced convection), for a period of 4 h. For comparison purposes, hollow fibers taken from the commercial hemodialyzer Fresenius F8HPS (Fresinius Medical Care AG and Co. KGaA; Bad Homburg, Germany) were also evaluated under the same conditions. Results showed that the MMM2 removed 367 mg of IS and 380 mg of pCS per square meter (m^2^) of the membrane, both of which are higher than the values obtained for the first-generation MMM1, 252 mg/m^2^ of IS and 160 mg/m^2^ of pCS. Furthermore, the removal of both PBUTs was higher in the MMM2 than obtained for the commercial hollow fiber which only removed 187 mg/m^2^ of IS and 225 mg/m^2^ of pCS. Due to the fact that, after the experiments, the authors found no trace of the PBUTs in the dialysate, they concluded that, in the MMM2, absorption was the mechanism responsible for the PBUTs removal and that the commercial membrane diffusion was the responsible mechanism [39].

With the aim of further reducing the inner diameter of the MMM2 fibers as well as increasing the K_UF_ towards achieving HFHD, a third generation of dual-layer PES/PVP-AC-based membranes, named MMM3, was developed by Kim et al. [40]. The authors were able to produce dual-layer MMM3 fibers with internal diameters of ~200 µm and a K_UF_ value of 174 mL/h/m^2^/mmHg, making them suitable for HFHD. The efficiency of the MMM3 in terms of PBUT removal was evaluated by subjecting the membranes to crossflow filtration of human plasma spiked with IS (40 mg/L) and HA (110 mg/L), at a TMP of 0 mmHg for 4 h, to promote diffusion transport. For comparison purposes, three different commercial hollow fiber membranes were also studied: (i) F8HPS (Fresinius Medical Care AG and Co. KGaA; Bad Homburg, Germany), (ii) FX1000 (Fresenius Medical Care AG and Co. KGaA; Bad Homburg, Germany), and (iii) Polyflux 2H (Baxter International, Inc.; Deerfield, IL, USA). Results showed that the MMM3 removed the highest amount of IS, reaching a value of 500 mg per m^2^ of the membrane, which was much higher than the amount of IS removed by the commercial membranes: 272, 377, and 370 mg of IS per m^2^ of membrane for the F8HPS, FX1000, and Polyflux 2H, respectively. Furthermore, no traces of IS were found in the dialysate compartment of the MMM3, indicating that the PBUT was adsorbed by the AC [40]. In terms of HA, the MMM3 was able to remove 2478 mg/m^2^ which is over 3 times higher than was seen for the first-generation PES/PVP-AC-based membranes (784 mg/m^2^) [38,40]. The removal of HA by the F8HPS, FX1000, and Polyflux 2H commercial membranes was >2600 mg/m^2^ which is higher than the value obtained for the MMM3 [40]. The rejection coefficient to HSA was 99% for the MMM3 and between 91 and 97% for the commercial membranes, indicating that the MMM3 is more efficient than the commercial membranes in terms of retaining protein [40].

The most recent generation of dual-layer PES/PVP-AC-based hollow fibers, MMM-OIF, developed by ter Beek at al. [41] introduced a new concept of circulation mode—the outside-in filtration (OIF) or extraluminal flow therapy, where the blood/plasma flows on the outside of the hollow fibers while dialysate flows in the lumen. In these membranes, the dense, thin, and particle-free layer is on the outside of the fiber while the inner side (lumen) is composed of the thicker and porous layer containing the AC particles. Because the blood flows on the outside of the hollow fibers, there is no direct contact with AC [41]. The membranes were evaluated in terms of K_UF_, protein leakage, and removal of HA and IS. For comparison purposes, hollow fibers taken from the commercial hemodialyzer, Polyflux 2H (Baxter International, Inc.; Deerfield, IL, USA), were also evaluated. The inner diameter of the MMM-OIF was 329 µm which is larger than the commercial membrane (218 µm) and the K_UF_ of the MMM-OIF, 100 mL/h/m^2^/mmHg, was lower than the value found for the commercial membrane (144 mL/h/m^2^/mmHg). Permeation experiments with the Polyflux 2H membranes were performed in the OIF mode as well as the conventional IOF mode, while the MMM-OIF was only tested in the OIF mode. The permeation studies of HA and IS were performed with human plasma spiked with HA (110 mg/L) and IS (40 mg/L) and at a TMP of 0 mmHg (diffusion only—no forced convection). Results showed that HA removal was 1466 mg/m^2^ for the MMM-OIF, 1436 mg/m^2^ for the commercial membrane used in OIF mode, and 1562 mg/m^2^ in the IOF mode, indicating that, in terms of removal of HA there was no significant difference between the MMM-OIF and the commercial membrane. Removal of HA by the MMM-OIF was attributed to adsorption by the AC particles as a very low amount of HA was detected in the dialysate, but the authors conclude that after 4 h of permeation, the AC became saturated as traces of HA were found in the dialysate. In terms of IS removal, the MMM-OIF removed 860 mg/m^2^ which is more than double the amount of IS removed by the commercial membrane which was 366 mg/m^2^ and 374 mg/m^2^ operating in OIF and IOF modes, respectively. The authors concluded that the highly efficient removal of IS by the MMM-OIF fibers is attributed to adsorption by the AC particles as no IS was found in the dialysate. Despite the promising results in terms of IS removal, two disadvantages of the MMM were identified: (i) the authors found that approximately 20% of total plasma proteins were adsorbed by the MMM-OIF, a very high value that will negatively impact further IS removal by reducing the accessibility of PUBTs to AC particles, and (ii) after 24 h of permeation, HSA was present in the dialysate of the MMMs while no trace of HSA was found in the dialysate of the commercial membrane [41]. The results obtained for the MMM-OIF hollow fibers are very promising and the authors have yet to test the OIF mode with forced convection which could further improve IS and HA removal; however, protein leaking must be carefully addressed.

Despite being very promising in terms of PBUT removal, the use of AC particles as a sorbent for blood-contacting devices continues to pose risks due to its poor biocompatibility [37]. Having this in mind, other researchers focused on developing membranes containing other particles known to absorb PBUTs, such as zeolites and metal-organic frameworks (MOFs) [42,43].

Zeolites are crystalline nanoporous aluminosilicates, which have been used as selective and efficient adsorbents in several adsorption separations and/or purification processes including drying of gases and liquids, air separation, and separation of linear hydrocarbons from branched hydrocarbons [44]. Having in mind the nanostructures structure of zeolites as well as their adsorptive properties, Lu et al. [45] studied the creatinine adsorption behavior of electrospun polyacrilonitirle-zeolite composites (30 wt.% P87-zeolite) and found that these were able to eliminate approximately 1 mg of creatinine per g of the membrane. P87 is a zeolite composed of a ratio of SiO_2_/Al_2_O_3_ of 87 [43]. Lu and Yeaow [43] fabricated a PES-zeolite (50 wt.% P87 zeolite) flat sheet MMM and studied its efficiency in terms of removal of IS. For comparison purposes, a pristine PES membrane was also prepared, and both were characterized in terms of IS removal and hydraulic permeability (Lp). To study the IS adsorption behavior, the membranes together with a sample of pure P87-zeolite were incubated separately, under static conditions, in aqueous solutions of IS (35 mg/L) for 3 h [43]. Due to the fact that the studies were not performed in the presence of plasma proteins, all of the IS was in the free or unbound form. The first conclusion was that the incorporation of P87-zeolite increased significantly the Lp from 0.52 g/m^2^/h/mmHg for the pristine PES membrane to 112 g/m^2^/h/mmHg for the PES-zeolite MMM. Scanning electron microscopy (SEM) images of the cross-section of the MMM show pores that cross the PES-zeolite membrane from the dialysate-contacting side to the blood-contacting surface which can explain the very high Lp value, while the pristine PES membrane exhibited a smooth and dense blood-contacting surface [43]. Other results showed that the pure P87-zeolite sample and the PES-zeolite membrane were able to absorb ~25% of IS from the aqueous solution, whereas the pristine PES membrane did not adsorb any. Furthermore, the PES-zeolite membrane was able to remove 550 µg of IS per g of the membrane [43]. Despite promising results, more studies are required to understand if the PES-zeolite membrane is also efficient in the presence of IS when it is in the bound form (in the presence of plasma proteins).

Metal-organic frameworks (MOFs) are a class of crystalline micro/mesoporous hybrid materials composed of metal ions or metal clusters interconnected by organic linkers [46]. Zeng et al. [42] investigated the adsorption performance of zirconium MOFs (Zr-MOFs) and found that they exhibited good performance in terms of adsorption of IS and HA. Three MMMs were prepared by incorporating up to 20 wt.% of three different Zr-MOFs: UiO-66, UiO-66-SO_3_H, and UiO-66-(COOH)_2_, with polylactide (PLA), rendering M-U, M-U-S, and M-U-C membranes, respectively. For comparison purposes, a pure PLA membrane was also prepared, and all membranes were characterized in terms of Lp and retention of bovine serum albumin (BSA). For all membranes, the adsorption of IS and HA was evaluated using solutions of each PBUT at a concentration of 500 mg/L dissolved in a saline solution (without plasma proteins), under static conditions, for 4 h. The M-U-S and pure PLA membranes were also evaluated under dynamic conditions in a circulatory ultrafiltration system for 3 h, using saline solutions containing each PBUT at a concentration of 500 mg/L [42]. The Lp of the MMMs, ~255 L/h/m^2^/bar, was significantly higher than the Lp of the pristine PLA membrane, 125 L/h/m^2^/bar. This increase was attributed to the larger membrane pore sizes formed upon the incorporation of the Zr-MOFs membranes. The retention of BSA was ~85% for the MMMs, which is above the required value, giving clear evidence of protein leaking. In terms of adsorption of IS and HA, results show that, under static conditions, all the MMMs were able to adsorb ~80% of the IS and HA, while the pure PLA membrane was only able to adsorb ~13% of both PBUTs [42]. Under dynamic conditions, the M-U-S membrane was able to remove more than 50% of IS and HA after 30 min of recirculation while the pristine PLA membrane removed less than 3% after 3 h of recirculation [42]. It can be concluded that the incorporation of Zr-MOFs into PLA membranes augments the adsorption of IS and HA and may be a promising approach towards HD membranes with enhanced removal of PBUTs. However, further studies are needed to understand if the same results are obtained when these PBUTs are present in plasma or blood where a large fraction will be bound to HSA.

## 3. Displacement-Based Approaches for the Removal of PBUTs

In this section, two displacement-based approaches being researched towards the enhanced removal of PBUTs are discussed: infusion of albumin displacers into the bloodstream of patients before or during HD sessions as well as the early stages of development of competitive binding membranes.

### 3.1. Infusion of Binding Competitors

HSA is the most abundant protein in blood plasma and, because of its extraordinary ligand binding capacity, is responsible for the transport of a wide variety of pharmaceutical drugs throughout the body [47,48]. As mentioned before, the HSA primary binding sites for the PBUTs IS, pCS, IAA and HA is Sudlow’s site II, whereas for CMPF is Sudlow’s site I [3,29]. The HSA-PBUT association constants (K_a_) for IS, pCS, IAA, HA, and CMPF are 0.98 × 10^5^ M^−1^, 1.0 × 10^5^ M^−1^, 2.1 × 10^5^ M^−1^, 0.1 × 10^5^ M^−1^, and 130.5 × 10^5^ M^−1^, respectively [49,50].

Considering that certain pharmaceutical drugs circulate in the blood bound to the same HSA primary binding sites as PBUTs and hence, when present in the same environment as PBUTs, compete for the same binding site, they are often referred to as competitive binding molecules or albumin displacers. When the association constant between the drug and HSA is larger than the value of K_a_ between HSA and the PBUT, the latter will tend to release itself from HSA and be replaced by the binding competitor. This in turn increases the percentage of the free (unbound) form of the PBUT which is readily removed by conventional HD membranes. A parallel phenomenon which should be considered is the allosteric mechanism, where a molecule binds to other binding sites of HSA causing the protein molecule to undergo modifications of its conformation which can lead to the release of the PBUTs [51,52]. This was first seen by Loor et al. [53] when trying to overcome the limitations of different methods available for the quantification of toxins in the plasma and using sodium octanoate as a binding competitor to separate IS and pCS from HSA.

**Figure 3:**
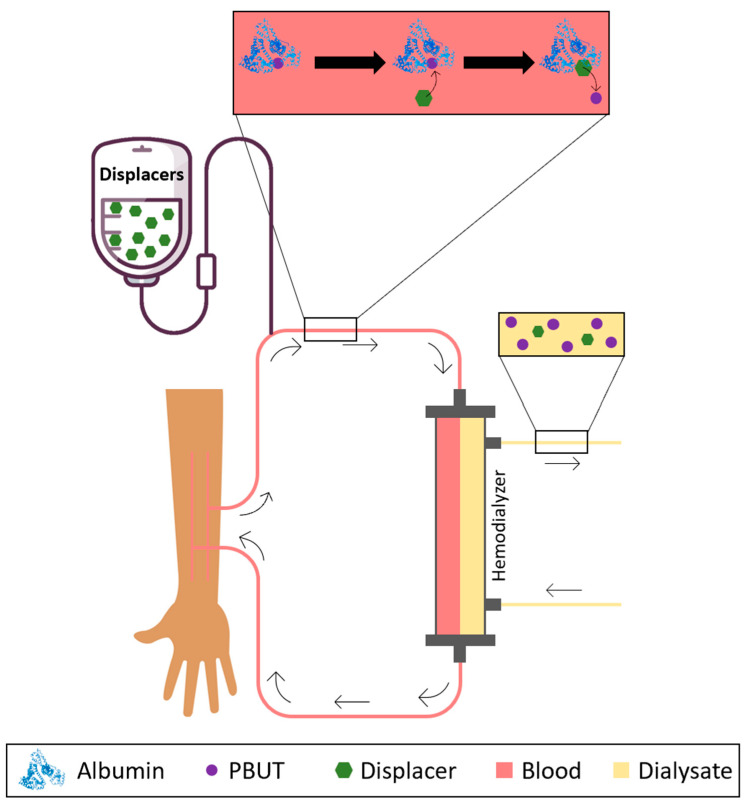
Schematical representation of the removal of PBUTs by HD after infusion of HSA displacers or binding competitors into the bloodstream.

Having this in mind, researchers have considered the possibility of infusing binding competitors or albumin displacers into the blood or plasma as a way of promoting the release of PBUTs from albumin and increasing the concentration of the free fraction which is then easily removed by HD membranes. Figure 3 schematically shows the concept behind the infusion of albumin displacers into the bloodstream of patients undergoing HD.

Tao et al. [54] studied the in vitro removal of IS using spiked HSA with IS (21.3 mg/L) under static and dynamic conditions in the presence of two different binding competitors: L-tryptophan (TRP) (weak displacer of IS, K_a_ = 1 × 10^4^ M^−1^) and docosahexaenoic acid (DHA) (strong displacer, K_a_ = 6.9 × 10^8^ M^−1^) [54]. In the experiments under static conditions, the spiked HSA-IS solutions were incubated with TRP and DHA, separately, for 4 h, in order to calculate the amount of IS displaced by each of the drugs. In experiments performed under dynamic conditions, the HSA-IS solution was circulated through a commercial HF hemodialyzer (F41S by Fresenius Medical Care; Waltham, MA, USA) for 10 min, after which the solution containing either TRP or DHA was administered to the reservoir containing the spiked plasma until a final concentration of 1 mmol/L was achieved. TRP or DHA was continuously infused into the blood flow circuit between the reservoir and the dialyzer until the end of the 4 h experiment at a rate of 50 µmol/min for TRP or 60 µmol/min for DHA. The feed and dialysate flow rates were 100 and 150 mL/min (counter-current, without recirculation) and the TMP was 0 mmHg (no forced convection). Results show that, under static conditions, the initial unbound fraction of IS was 8% and after being incubated for 4 h with TRP and DHA had increased to 11 and 25%, respectively [54]. Results from the study performed under dynamic conditions showed that the removal of IS was 19 and 28% for TRP and DHA, respectively [54]. DHA showed better results, confirming that is a stronger binding competitor compared to TRP and can successfully displace IS from HSA. As a negative control, phosphate-buffered saline (PBS), which does not contain displacers, was infused through the same hemodialyzer and the removal was only 10% which is approximately the same amount of the unbound fraction present at the beginning of the experiment [54].

Tao et al. [51], performed, ex vivo experiments, using human plasma and whole blood spiked with IS, IAA, HA, and pCS, for the evaluation of different binding competitors: ibuprofen (IBF), furosemide (FUR), and TRP. The biological samples used were: (i) plasma from ESRD patients (ii) plasma from a healthy population, and (iii) whole blood from a healthy population. In the experiments under static conditions, the uremic plasma was incubated with IBF (1 mmol/L), FUR (0.18 mmol/L), and TRP (1 mmol/L), separately, for 4 h at 37 °C, to evaluate the displacement of IS, pCS, and HA [51]. The authors also evaluated under static conditions and using healthy plasma spiked with IS at a concentration of 32 mg/L, the dose-response to IBF (0 to 931 µmol/L) as well as the effect of using a mixture of IBF and FUR ([IBF] 0 to 931 µmol/L, and [FUR] fixed at 0 or 182 µmol/L) [51]. The latter study (was carried out to evaluate if the infusion of FUR together with IBF would boost the IS displacement effect of IBF when given simultaneously, suggesting additive or synergetic effects of these two binding competitors. The authors also performed an in vitro study under dynamic conditions, using hollow fibers taken from the Optiflux^®^ F160NR hemodialyzer (Biotechnology Research Group, Dialyzer R&D; Ogden, UT, USA), with a total surface area of 0.039 m^2^. In this study, the removal of IS (32 mg/L), IAA (2.6 mg/L), and HA (71.6 mg/L) from healthy whole blood was evaluated by a single-pass through the hemodialyzer (without recirculation), with blood and dialysate flow rates of 12.5 and 25 mL/min (counter-current), respectively, and with a TMP of 0 mmHg (no forced convection). A combination of IBF/FUR (IBF, 116 mmol/L / FUR, 23 mmol/L) or simply TRP (146 mmol/L) was infused at a rate of 70 μL/min and 138–150 μL/min, respectively [51].

Results showed that under static conditions, the free fraction of IS and pCS increased in the presence of FUR, TRP, and IBF, by a factor of 1.3, 2.0, and 3.0, respectively, indicating that IBF has the highest binding affinity towards IS and pCS. For HA the highest increase of the free fraction was found in the presence of FUR, followed by IBF, and TRP did not show differences when compared with the control [51]. The evaluation of the dose-response effect of IBF revealed that the increase in IBF concentration resulted in an increase in IS displacement and the addictive or synergistic effect of using IBF and FUR as binding competitors was verified [51]. Under dynamic conditions, results showed that the combinations of IBF/FUR increased the removal of IS by a factor of 3 (from 6 to 18%) and IAA by a factor of 2 (from 17 to 35%). In the presence of TRP, the removal of IS increased from 6 to 11% (1.6×) and the removal of IAA increased from 17 to 27% (1.6×). None of the binding competitors showed a significant impact on the removal of HA. The authors concluded that this approach has the potential to be applied in current clinical HD sessions because all the binding competitors are FDA-approved and none were administered at toxic concentrations [51]. However, it is important to note that TRP, which was used in these studies, is known to be metabolized into an indole, and ultimately converted to IS, which is exactly one of the target PBUTs and therefore should not be administrated into the bloodstream of ESRD patients.

In 2019, the first proof-of-concept clinical study was performed to explore the potential of using IBF as an albumin displacer to increase the depletion of IS and pCS in ESRD patients during HD sessions [55]. The study was performed on 18 ESRD patients, with no residual urine production, during a 4 h-long therapy session to whom heparin was administrated prior to the beginning of the HD session. HFHD hemodialyzers Hemoflow F80A (Fresenius Medical Care; Waltham, MA, USA) were used and the blood and dialysate flow rates were set to 300 and 500 mL/min, respectively. The HD session was divided into three phases: (i) pre-infusion (0 to 20 min of HD session); (ii) IBF infusion (21 to 40 min of HD session); and (iii) post-infusion (41 to 240 min of HD session). The only intervention during the 4 h was during the infusion phase, where an IBF solution (3200 mg/L) was administered to the arterial blood line for 20 min at a rate of 12.5 mL/min (40 mg of IBF/min). The three phases were analyzed in terms of IS and pCS removal [55] and the results showed that during the second phase (infusion of IBF) the clearance of IS and pCS increased from 6 to 20 mL/min and from 4 to 15 mL/min, respectively [55]. During the post-IBF-infusion phase, the clearance of IS and pCS decreased to values similar to those seen in the pre-IBF-infusion. The authors conclude that IBF improves the removal of IS and pCS in ESRD patients undergoing a single HFHD session, but the competitive action of IBF occurs primarily during the infusion phase and that better results could be obtained if IBF is administered continuously during the entire HD session [55]. IBF is a dialyzable compound, that easily crosses the hollow fiber membranes in the hemodialyzer. This means that, when not being infused, IBF is quickly eliminated during the HD session, reducing the possibility of the displacement of the HSA-PBUTs complex to occur. Hence, there is a need to infuse IBF throughout the HD session which could prove to be a limitation of this approach. Other compounds with competitive properties (on their own or mixed with others) and other displacer-based approaches should be explored [55].

Instead of using pharmaceutical drugs, Li, et al. [52] studied the competitive binding properties of natural herbal medicinal products, known to have standardized active ingredients, towards the enhanced removal of IS and pCS. Danhong (DHI) is a natural product commonly used intravenously in traditional Chinese medicine for the treatment of cardiovascular and cerebrovascular diseases and has also shown beneficial effects in confirmed cases of deterioration of kidney function [56]. DHI is extracted from the rhizome of Salvia miltiorrhiza Bunge (*Labiatae*) and the dry flower of *Carthamus tinctorius* L. (*Asteraceae*). The main bioactive compounds of DHI are salvianolic acid-based, including lithospermic acid (LA), salvianolic acid A (SaA), tanshinol (danshensu, DSS), caffeic acid (CA), salvianolic acid B (SaB), protocatechuic aldehyde (PA) and rosmarinic acid (RA) [52]. RA, SaB, and LA are reported to be strong protein-binding ligands, with binding percentages for HSA between 92 and 99% [57]. Li, et al. [52] studied the potential of DHI and the salvianolic acid LA as binding competitors and studied the removal of PBUTs in in vitro and in vivo rat models. In vitro studies were performed with healthy rat plasma spiked with IS (50 mg/L) and pCS (50 mg/L), followed by an infusion of DHI, LA, and IBF, at a later stage. During the first two hours, the spiked plasma was flown through a dialysis probe (Microbiotech/se AB; Stockholm, Sweden) with a feed flow rate of 2 µL/min (single-pass HD), and during the last two hours, the spiked plasma containing each of the binding competitors at different concentrations (50, 200 or 400 µM) was passed through the same hemodialyzer (single-pass). For comparison purposes, IBF was also studied at the same concentrations as well as 1000 µM, under the same circulation conditions. Results showed that DHI (400 µM) improved the removal of IS and pCS by 99 and 142%, respectively. The salvianolic acid, LA (400 µM), increased the removal of IS and pCS by 197 and 198%, respectively, and infusion of IBF (400 µM) did not affect IS or pCS removal. Finally, it was concluded that, because the primary binding site of IS and pCS is different from that of LA, the displacement of HSA from the PBUTs is due to allosteric mechanisms rather than direct binding competitions [52]. In the in vivo rat model, rats with very limited or no kidney function were subjected to microdialysis for 4 h, using a dialysis probe (Microbiotech/se AB; Stockholm, Sweden) and a blood flow rate of 2 µL/min. Conventional HD was performed for two hours after which DHI or LA (24.7 mg/kg) was infused at concentrations of 4 and 25 mL/kg, respectively. HD was then continued for an additional 2 h [52]. Results showed that DHI increased the removal of IS and pCS by 136 and 272%, respectively and LA increased the removal of IS and pCS by 120 and 128%, respectively. Higher removal rates of IS and pCS were found when LA was infused in comparison to the first two hours where no binding competitor was present [52]. Improved results are obtained when a cocktail of molecules is administered, rather than used separately. This indicates an additive or synergistic effect of salvianolic acid-based compounds, which was also suggested by Tao et al. [51] when using a mixture of IBF and FUR simultaneously as binding competitors.

Intravenous lipid emulsions (ILEs) have been used in various clinical fields ranging from parental nutrition to resuscitation from drug overdoses. After being injected, the free fatty acids (FFAs) in the emulsion are released and can bind to nine different binding sites of HSA (FA1 to FA9). Shi et al. [58] studied the binding of ILEs compounds to HSA in the presence of PBUTs. In vitro binding and HD studies were performed using HSA solutions and uremic rat models were used for the in vivo experiments. Oleic acid (OA) and linoleic acid (LLA), which mimic conventional lipid emulsions, and eicosapentaenoic acid (EPA) and docosahexaenoic acid (DHA) which mimic omega-3 fish oil lipid, were the ILEs used in the in vitro assays. HSA solutions (40 g/L) were spiked with CMPF (61.2 mg/L), pCS (37.6 mg/L), IS (32.0 mg/L) and IAA (2.6 mg/L), separately and the competitive binding effect of the four different FFAs was evaluated by adding them to the spiked HSA solutions at concentration: OA (0.01 to 4.8 mmol/L), LLA (0.01 to 4.8 mmol/L), EPA (0.01 to 10.0 mmol/L) and DHA (0.01 to 10.0 mmol/L). Results show that OA and LAA significantly increased the free fraction of all the PBUTs, and CMPF was the one where the effect was highest, as it was displaced by 95%, followed by pCS, IS, and IAA. It was also concluded that OA and LA were much stronger displacers when compared to EPA and DHA. The in vitro HD study was performed using a polysulfone-based hemodialyzer suitable for small animals. The feed and dialysate flow rates were 10 and 6 mL/min, respectively, (counter-current mode), and the TMP was adjusted to 0 mmHg (no forced convection). An HSA solution (40 g/L) was spiked with CMPF, pCS, IS, and IAA, at concentrations of 61.2, 37.6, and 32.0 mg/L, respectively, and was circulated through the dialyzer for 10 min. Then, a mixture of LLA and OA (LLA:OA ratio 2:1) was infused continuously in the feed inlet to a final concentration of 1 mmol/L. As a negative control, the same procedure was performed using PBS (without FFAs). Results show superior removal of PBUTs when FFAs were infused. The removal of CMPF increased from 0 to 14%; pCS increased from 8 to 28%; IS increased from 12 to 35% and IAA increased from 15 to 40% [58]. In the first in vivo study, a rat model (with ESRD) was used, and the commercial ILE Intralipid^TM^ was infused into the bloodstream of rats at a concentration of 3 mL/kg (per animal weight). A control group composed of rats with ESRD was infused with PBS. Blood samples were collected before and after the administration of ILE, and the binding profile between PBUTs and HSA was registered over time. Results show that after 30 min of administration of Intralipid^TM^, the concentrations of the free forms of CMPF, pCS, IS and IAA present in the rat’s blood had significantly increased, reaching the highest value after 60 min of administration. However, after 210 min of infusion, the concentrations of the free forms were lower than the initial values. The authors concluded that FFA has the potential to displace PBUTs from HSA, but that the effect is reversible after a short period [58]. The second in vivo study was performed for 3 h using the same polysulfone-based hemodialyzer, the blood and dialysate flow rates were 1 and 5 mL/min, respectively (counter-current mode, without recirculation), and the TMP was adjusted to 0 mmHg (no forced convection) [58]. After 10 min of HD, Intralipid^TM^ (3 mL/kg of animal weight) was infused continuously until the end of the HD session while PBS was infused into the control group (rats with ESRD). The reduction ratio of pCS, IS and IAA was 3.3-, 2.1- and 1.7 times higher for the rats infused with Intralipid^TM^ than for the control group. The authors concluded that infusion of lipid solutions can lead to superior removal of PBUTs during HD sessions but advise that further studies are required to understand the potential side effects of long-term treatment with Intralipid^TM^ [58].

### 3.2. Competitive Binding Membranes

**Figure 4:**
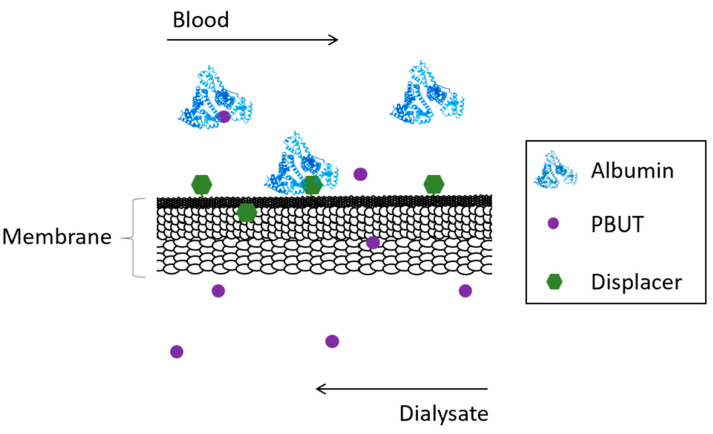
Schematical representation of the removal of PBUTs by competitive binding membranes.

Competitive membranes are membranes which combine the characteristic filtration properties of the pure polymer membrane coupled with the ability to displace albumin from bound toxins by competitive binding. The competitive binding membranes are described as monophasic hybrid cellulose acetate/silica-based (CA/SiO_2_-based) membranes where the displacer is covalently bound to the polymer matrix of the membrane reducing drastically the risk of having it be leached into the blood as was seen for the adsorptive MMMs. Figure 4 schematically shows the concept behind competitive binding membranes.

Mendes et al. [59] were the first to develop CA/SiO_2_ monophasic hybrid membranes (MHMs) characterized by the covalent bonding between the inorganic (silica) and organic (cellulose acetate) elements, by an innovative method which couples phase inversion and sol-gel techniques. In contrast to the adsorptive MMMs described in Section 2, where the physical incorporation of inorganic particles into the polymer matrix renders membranes with two distinct phases (clusters of particles dispersed in a continuous polymer matrix) [37,38,39,40,41,42,43], the MHMs exhibit only one phase where silica and CA are covalently bonded eliminating the risk of particles leaching from the membranes and consequent release into the bloodstream of the patients.

Membranes containing up to 40 mol% SiO_2_ covalently bonded to CA were fabricated by reacting the silica precursor tetraethyl orthosilicate (TEOS) with CA under acidic conditions [59]. Six integral asymmetric membranes containing 0, 10, 15, 20, 30, and 40 mol% were prepared and the covalent bonding between SiO_2_ and CA was confirmed by Fourier transform infrared spectroscopy in attenuated total reflection mode (ATR-FTIR) with identification of the peak corresponding to the Si-O-C bond. The permeation performance of the MHMs was evaluated in terms of Lp and MWCO and results showed that the incorporation of 15 mol% silica increased the Lp to 59 kg/h/m^2^/bar, a value much higher than was found for the pristine CA membrane, 32 kg/h/m^2^/bar. Furthermore, the MWCO also increased from 54 kDa to 111 kDa for the pristine CA and 15 mol% silica membranes, respectively. For higher contents of SiO_2_, the Lp value decreased to lower values than was seen for the pure CA membrane [59].

Faria et al. [60], performed further studies on CA/SiO_2_ membranes containing 0, 5, 11, and 18 wt.% of SiO_2_ in terms of hemocompatibility and permeation performance. Hemocompatibility assays revealed that all the membranes were non-hemolytic and that the CA/SiO_2_ membranes presented lower thrombosis degrees and levels of platelet adhesion and activation when compared to the pristine CA membrane, indicating that the incorporation of silica enhances hemocompatibility making MHMs good candidates for blood-contacting applications. In terms of permeation performance, the average Lp of the membranes containing silica was 49 L/h/m^2^/bar which is higher than pristine CA membrane by a factor of 2. Furthermore, all monophasic hybrid CA/SiO_2_ membranes fully permeated urea (a surrogate marker of free water-soluble LMWMs) and retained ~100% albumin [60].

Considering the chemical versatility and high reactivity of primary amines (-NH_2_), Andrade et. al. [61] developed a new group of MHMs, named CA/SiO_2_/SiO_1.5_-(CH_2_)_3_NH_2_ membranes, by incorporation of a second silica precursor, (3-aminopropyl)-triethoxysilane (APTES), into the CA/SiO_2_-based membranes [61]. This functionalization can have the potential for chemical, pharmaceutical, and biomedical applications [61]. MHM with a fixed content of 5 wt.% SiO_2_ (derived from TEOS) and contents of SiO_1.5_-(CH_2_)_3_NH_2_ (derived from APTES) varying between 0 and 50 mol% were prepared and characterized in terms of permeation performance [61]. The incorporation of 10 mol% SiO_1.5_-(CH_2_)_3_NH_2_ increased the Lp values from 23 to 69 kg/h/m^2^/bar and for membranes with the content of SiO_1.5_-(CH_2_)_3_NH_2_ higher than 20 mol% the Lp values were lower than the Lp of the pure CA membrane [61].

Janeca et al. [62], studied the permeation performance of the CA/SiO_2_/SiO_1.5_-(CH_2_)_3_NH_2_ membrane containing 5 wt.% SiO_2_ and a molar ratio of SiO_2_/SiO_1.5_-(CH_2_)_3_NH_2_ of 80:20 under dynamic conditions in a lab-scale hemodialysis circuit. For comparison purposes, a pure CA membrane was characterized in the same set-up under the same conditions. Results showed that the incorporation of SiO_2_ and SiO_1.5_-(CH_2_)_3_NH_2_ increased the Lp from 28 (pure CA membrane) to 51 kg/h/m^2^/bar and the MWCO from 18 (pure CA membrane) to 25 kDa. Additionally, the CA/SiO_2_/SiO_1.5_-(CH_2_)_3_NH_2_ membrane fully permeated urea, creatinine, and uric acid (LMWMs) and retained 100% albumin [62].

The possibility to covalently bond two different silica precursors, TEOS and APTES, to the CA matrix membrane presents an advantage as it eliminates the risk of leaching the inorganic materials into the bloodstream of the patients [59,60,61,62]. Moreover, it introduces a novel and highly efficient method of covalently incorporating a wide range of different compounds into CA membranes. Silica precursors can be functionalized with other molecules, such as polyureas and azo dyes [63,64,65] prior to being reacted with CA by the sol-gel reaction. Very recent studies show the successful modification of TEOS and APTES with IBF a well-known HSA displacer [66] and it is envisioned that both precursors can be functionalized with other drugs such as FUR and TRP. This opens the door for a huge number of opportunities namely the fabrication of new competitive binding membranes where the albumin displacer is covalently bonded to the polymer matrix of the membrane. Lopes et al. [66], recently reported the synthesis of two new silica precursors, TEOS-IBF and APTES-IBF as well as the fabrication of integral asymmetric CA/SiO_2_/IBF MHMs containing up to 15 wt.% of IBF and in which the silica precursors are covalently bonded to the CA polymer [66]. It is expected that when the blood of uremic patients reaches the hemodialyzer and encounters the membranes, the displacers will compete with PBUTs for the same HSA binding site resulting in an increase in free PBUTs, which are easily dialyzed by the CA/SiO_2_/IBF membranes. If this approach has the success that the authors expect, competitive membranes may be more advantageous than the infusion of large doses of drugs into the bloodstream and will present much fewer risks to ESRD patients.

## 4. Conclusions

The removal of PBUTs from patients suffering from CKD and ESRD remains a huge challenge and, in the last few years, has become one of the main targets of clinical physicians, membrane scientists as well as the general renal community. In this review, adsorption- and displacement-based approaches targeting the removal of PBUTs are described and it is suggested that they may present an important step in the advancement of renal replacement therapies.

Adsorptive membranes, characterized by the incorporation of particles such as activated carbon, zeolites, or MOFs, into the polymer matrix of HD membranes, exhibit better results in terms of the removal of PBUTs when compared to current HD membranes. However, there are concerns regarding the hemocompatibility of the adsorptive materials and further studies must be performed to understand their effect on vital blood components such as plasma proteins, HSA, platelets, etc., as well as the quantification of plasma proteins that are being absorbed by these particles.

The infusion of pharmaceutical drugs or natural bioactive compounds with a high affinity for HSA are being used to displace PBUT-HSA complexes, increasing the concentration of the free fraction of PBUTs which is cleared using commercial HD membranes. Even though several competitive binders such as IBF and TRP have shown great potential towards depletion of PBUTs, extensive studies regarding the toxicity and long-term effects of administering these drugs to patients three times a week for the rest of their lives are needed. Furthermore, the impact of the displacers on other pharmaceutical drugs which ESRD patients regularly rely on, such as anticoagulants to minimize the risk of coagulation during HD sessions, must be evaluated.

Competitive binding membranes characterized by integral asymmetric monophasic hybrid films to which albumin displacers, such as IBF, are covalently bonded, are still in the early stages of evaluation, and further studies are needed to confirm their capability of removing PBUTs. Nevertheless, they present a new trend for the development of smart, toxin-competitive HD membranes.

The approaches described in this review seem promising toward the depletion of IS, pCS, HA, IAA, and CMPF, but these are only a small fraction of the total number of PBUTs identified to date and therefore additional studies are needed to evaluate their potential of eliminating other PBUTs. Overall, adsorption- and displacement-based approaches have proved to be successful strategies towards the removal of PBUTs, but further research is required to understand the associated risks to each technology.

## Data Availability

Not applicable.

## Abbreviations:

AC—activated carbon; OA—oleic acid; APTES—(3-aminopropyl)-triethoxysilane; ATR-FTIR—Fourier transform infrared spectroscopy in attenuated total reflection mode; BSA—bovine serum albumin; CA—cellulose acetate; CKD—chronic kidney disease; CMPF—3-carboxyl4-methil-5-propyl-furanpropionic acid; CTA—cellulose triacetate; DHA—docosahexaenoic acid; DHI—Danhong injection; DSS—danshensu = tanshinol; EPA—eicosapentaenoic acid; ESRD—end stage renal disease; EUTox—European Uremic Toxin work group; FDA—Food and Drug Administration; FA—fatty acid; FFA—free fatty acid; FUR—furosemide; HA—hippuric acid; HCO-HD—high cut-off hemodialysis; HD—Hemodialysis; HFHD—high flux hemodialysis; HSA—human serum albumin; IAA—indole-3-acetic acid; IBF—ibuprofen; ILE—intravenous lipid emulsion; IOF—inside-out filtration; IS—Indoxyl sulphate; K_UF_—ultrafiltration coefficient; LA—lithospermic acid; LFHD—low flux hemodialysis; LLA—linoleic acid; LMWM—free water-soluble low molecular weight molecule; Lp—hydraulic permeability; MCO-HD—medium cut-off hemodialysis; MHM—monophasic hybrid membranes; MMM—mixed matrix membrane; MOF—metal-organic framework; MW—molecular weight; MWCO—molecular weight cut-off; OA—oleic acid; OIF—outside-in filtration; PA—protocatechuic aldehyde; PBS—phosphate-buffered saline; PBUT—protein-bound uremic toxin; pCS—p-cresyl sulphate; PES—polyesthersulfone; PLA—polylactide; PS—polysulfone; PVP—polyvinylpyrrilidone; RA—rosmarinic acid; RRT—renal replacement therapy; SaA—salvianolic acid A; SaB—salvianolic acid B; SEM—scanning electron microscopy; TEOS—tetraethyl orthosilicate; TMP—transmembrane pressure; TRP—L-tryptophan; UT—uremic toxin; β_2_m—β_2_-microglobulin
